# Long-term prognosis in Takotsubo syndrome compared to heart failure: observations from a global federated research network

**DOI:** 10.1093/eschf/xvag065

**Published:** 2026-02-27

**Authors:** Enrico Tartaglia, Muath Alobaida, Tommaso Bucci, Michele Rossi, Amir Askarinejad, Ho Man Lam, Mert Kaskal, Andrea Galeazzo Rigutini, Giuseppe Boriani, Gregory Y H Lip

**Affiliations:** Liverpool Centre for Cardiovascular Science at University of Liverpool, Liverpool John Moores University and Liverpool Heart and Chest Hospital, 6 West Derby Street, Liverpool, L69 7TX, UK; Cardiology Division, Department of Biomedical, Metabolic and Neural Sciences, Italy University of Modena and Reggio Emilia, Policlinico di Modena, Modena, Italy; Liverpool Centre for Cardiovascular Science at University of Liverpool, Liverpool John Moores University and Liverpool Heart and Chest Hospital, 6 West Derby Street, Liverpool, L69 7TX, UK; Department of Basic Science, Prince Sultan Bin Abdulaziz College for Emergency Medical Services, King Saud University, Riyadh, Saudi Arabia; Liverpool Centre for Cardiovascular Science at University of Liverpool, Liverpool John Moores University and Liverpool Heart and Chest Hospital, 6 West Derby Street, Liverpool, L69 7TX, UK; Department of Clinical Internal, Anesthesiologic and Cardiovascular Sciences, Sapienza University of Rome, Rome, Italy; Liverpool Centre for Cardiovascular Science at University of Liverpool, Liverpool John Moores University and Liverpool Heart and Chest Hospital, 6 West Derby Street, Liverpool, L69 7TX, UK; Department of Life, Health & Environmental Sciences, University of L’Aquila, L’Aquila, Italy; Internal Medicine and Nephrology Division, ASL1 Avezzano-Sulmona-L’Aquila, San Salvatore Hospital, L’Aquila, Italy; Liverpool Centre for Cardiovascular Science at University of Liverpool, Liverpool John Moores University and Liverpool Heart and Chest Hospital, 6 West Derby Street, Liverpool, L69 7TX, UK; Rajaie Cardiovascular Medical and Research Institute, Iran University of Medical Sciences, Tehran, Iran; Liverpool Centre for Cardiovascular Science at University of Liverpool, Liverpool John Moores University and Liverpool Heart and Chest Hospital, 6 West Derby Street, Liverpool, L69 7TX, UK; Liverpool Centre for Cardiovascular Science at University of Liverpool, Liverpool John Moores University and Liverpool Heart and Chest Hospital, 6 West Derby Street, Liverpool, L69 7TX, UK; Department of Pharmacology, School of Medicine, Marmara University, Istanbul, Turkey; Liverpool Centre for Cardiovascular Science at University of Liverpool, Liverpool John Moores University and Liverpool Heart and Chest Hospital, 6 West Derby Street, Liverpool, L69 7TX, UK; Internal, Vascular and Emergency Medicine—Stroke Unit, University of Perugia, Perugia, Italy; Cardiology Division, Department of Biomedical, Metabolic and Neural Sciences, Italy University of Modena and Reggio Emilia, Policlinico di Modena, Modena, Italy; Clinical and Experimental Medicine PhD Program, University of Modena and Reggio Emilia, Modena, Italy; Liverpool Centre for Cardiovascular Science at University of Liverpool, Liverpool John Moores University and Liverpool Heart and Chest Hospital, 6 West Derby Street, Liverpool, L69 7TX, UK; Department of Clinical Medicine, Aalborg University, Aalborg, Denmark; Department of Cardiology, Lipidology and Internal Medicine, Medical University of Bialystok, Bialystok, Poland

**Keywords:** Takotsubo syndrome, Heart failure, Heart failure Systolic, Heart failure Diastolic, Cardiovascular outcomes

## Abstract

**Introduction:**

To compare long-term outcomes of patients with Takotsubo syndrome (TTS) and heart failure (HF).

**Methods:**

This retrospective observational study used the TriNetX global federated research network. Adult patients (≥18 years) discharged with a diagnosis of TTS (ICD-10-CM I51.81) or HF (I50.x) between 2018 and 2022 were identified. Primary outcomes were 3-year risk of all-cause death, major adverse cardiovascular events (MACE; myocardial infarction or ischaemic stroke), and acute HF. Secondary outcomes included myocardial infarction, ischaemic stroke, ventricular arrhythmias (ventricular tachycardia), malignant arrhythmias (ventricular fibrillation or cardiac arrest), and new-onset atrial fibrillation (AF). Cox proportional hazards models estimated hazard ratios (HRs) with 95% confidence intervals (CIs) before and after 1:1 propensity score matching (PSM). Subgroup analyses were performed by HF phenotype, age (≥65 vs <65 years), and mental health status.

**Results:**

The study included 2240 patients with TTS (mean age 62.6 ± 17.3 years; 73.7% female) and 265 564 patients with HF (69.3 ± 14.7 years; 45.8% female). After PSM, TTS was associated with a lower risk of acute HF (HR 0.622, 95% CI 0.539–0.717), ventricular arrhythmias (HR 0.637, 95% CI 0.441–0.919), malignant arrhythmias (HR 0.656, 95% CI 0.571–0.754), new-onset AF (HR 0.672, 95% CI 0.517–0.875), and myocardial infarction (HR 0.818, 95% CI 0.687–0.974), with no significant differences in the remaining outcomes. Differences were greater when TTS was compared with heart failure with reduced ejection fraction.

**Conclusions:**

TTS is associated with lower risk of adverse events than HF. Further research is needed on mental health in its pathogenesis and prognosis.

## Graphical Abstract

**Graphical Abstract:**
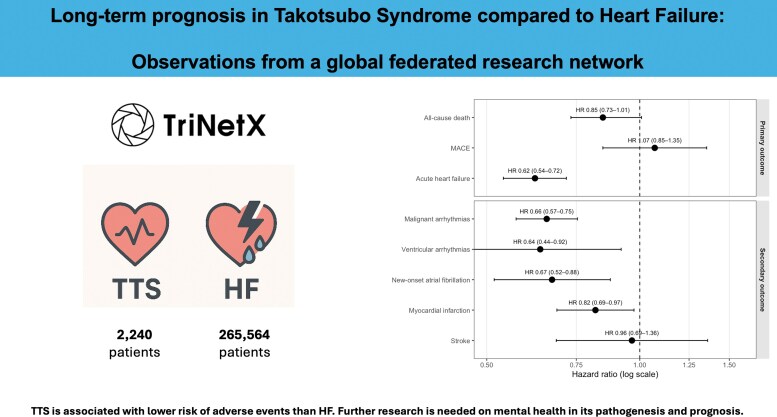
Adjusted hazard ratios for long-term clinical outcomes in 2,240 patients with takotsubo syndrome and 265,564 with heart failure from a global federated research network. TTS was associated with lower risks of acute heart failure and arrhythmic events, with no significant difference in all-cause mortality.

## Introduction

Takotsubo syndrome (TTS), also known as stress cardiomyopathy or ‘broken heart syndrome’, is an acute and transient cardiac condition first described in Japan in the 1990s.^1^ It is typically characterized by the sudden onset of chest pain and electrocardiographic changes that closely mimic acute coronary syndrome, usually in the absence of obstructive coronary artery disease.^2,3^ The hallmark feature of TTS is a reversible regional systolic dysfunction of the left ventricle, most often involving the apical and midventricular segments, resulting in the distinctive ‘ballooning’ appearance reminiscent of the Japanese octopus trap, or *Takotsubo*.^4–6^

Although its clinical presentation can be potentially life-threatening, TTS has traditionally been considered a benign and self-limiting condition.^7,8^ It predominantly affects postmenopausal women and is frequently precipitated by emotional or physical stress, although a substantial proportion of cases occur without an identifiable trigger.^2,9^ Despite ongoing research, the underlying pathophysiology remains only partially understood, with prevailing hypotheses focusing on catecholamine-mediated myocardial stunning and dysregulation of the brain–heart axis.^10,11^

Heart failure (HF), predominantly reflecting reduced ejection fraction owing to the prevailing systolic dysfunction in TTS, is among the most frequent complications during the acute phase of the syndrome, with clinical manifestations ranging from mild pulmonary congestion to cardiogenic shock.^12^ This overlap has led to increasing interest in the relationship between TTS and the broader HF spectrum—a syndrome classically defined by chronic progression, recurrent hospitalizations, and poor long-term outcomes,^12^ thereby establishing HF as a pragmatic comparator for contextualizing the clinical burden following TTS.

Despite their shared features, TTS and HF differ markedly in pathogenesis and natural history, with the former being acute and typically reversible, and the latter chronic and progressive. Whether these differences are reflected in distinct long-term outcomes remains unclear.

The aim of this study was to compare the long-term outcomes of patients with TTS to those with HF, with particular attention to reduced and preserved ejection fraction phenotypes, using data from a large global federated research network.

## Methods

### Study design

This retrospective observational study was conducted using the TriNetX Research Network (TriNetX, Cambridge, MA, USA), a global federated health research network that provides access to anonymized electronic medical records from over 130 healthcare organizations, including academic medical centers, community hospitals, and outpatient clinics. The network includes more than 300 million individuals, primarily in the USA.

Available data include demographics, diagnoses coded using the International Classification of Diseases, Tenth Revision, Clinical Modification (ICD-10-CM) codes, and medications coded with Veteran Affairs (VA) codes or RxNorm classification systems, depending on the contributing institution.

Further information about the network is available online (https://trinetx.com/about-trinetx/).

TriNetX operates in compliance with the Health Insurance Portability and Accountability Act (HIPAA) and United States (US) federal law. All patient data are de-identified in accordance with HIPAA’s Privacy Rule, and no patient-level identifiers are accessible. Access to the data is governed through formal data-sharing agreements with participating institutions. As a federated network using only de-identified data, studies conducted within TriNetX do not require approval from institutional review boards or informed consent.

Additional methodological details are provided in the Supplementary material.

### Cohort

The searches on the TriNetX online research platform were performed on 16 January 2026, using the Global Collaborative Network. Based on recorded ICD-10-CM, patients aged ≥18 years were included if they had a diagnosis of TTS (ICD-10-CM: I51.81) or HF (ICD-10-CM: I50.x).

Patients with concurrent diagnoses of myocardial infarction (ICD-10-CM: I21.x) were excluded from the TTS cohort to minimize diagnostic overlap. To ensure mutually exclusive comparator groups, patients with TTS were excluded from the HF cohort. Conversely, the presence of HF was not an exclusion criterion from the TTS cohort, as HF is often a clinical manifestation of TTS.

The index event was defined as the first recorded diagnosis of TTS or HF associated with a hospital discharge (CPT: 1013682) between 1 March 2018, and 1 March 2022, to reflect contemporary clinical practice and to ensure a minimum of 3 years of follow-up for all patients. At the time of the search, data from 148 participating healthcare organizations, exclusively located in the USA, were available for patients who met the study inclusion criteria. The construction of each cohort and the corresponding ICD-10-CM codes for inclusion and exclusion are reported in Supplementary Table S1. To further enhance transparency and reproducibility, a detailed cohort construction flowchart illustrating inclusion and exclusion criteria and the handling of overlapping ICD-10 diagnoses is provided in Supplementary Figure S1.

### Outcomes

The primary outcomes were the 3-year risk of all-cause death, major adverse cardiovascular events (MACE, defined as the composite of myocardial infarction and ischaemic stroke), and acute heart failure.

Secondary outcomes included the individual components of the MACE, ventricular arrhythmias, categorized as ventricular tachycardia (VT), malignant arrhythmias (defined as a composite of ventricular fibrillation and cardiac arrest)—and new-onset atrial fibrillation (AF).

For all outcomes, patients with a history of the event of interest before the observation window were excluded. Each outcome was assessed with follow-up beginning on day 31 after the index date to avoid capturing events related to the index hospitalization. Events were identified using ICD-10-CM codes (see Supplementary Table S2 for the full code list).

### Statistical analysis

Baseline characteristics of patients with TTS and those with HF were compared and balanced using logistic regression and propensity score matching (PSM) at a 1:1 ratio. Matching was performed using the greedy nearest neighbour method, employing a caliper of 0.1 pooled standard deviations, without replacement. The balance of demographics and clinical variables between the groups was assessed using Absolute Standardized Mean Differences (ASD), with an ASD of less than 0.1 indicating adequate balance. Density plots of the propensity score distributions were used to evaluate the quality of matching.

The following covariates were included in the PSM model: age, sex, ethnicity (e.g. Hispanic or Latino), hypertension, diabetes mellitus, dyslipidemia, obesity, ischaemic heart disease, prior myocardial infarction, peripheral artery disease, cerebrovascular disease, pulmonary embolism, atrial fibrillation, chronic kidney disease, left ventricular ejection fraction (LVEF), and cardiovascular medications (beta-blockers, ACE inhibitors, angiotensin receptor blockers, angiotensin receptor–neprilysin inhibitors, mineralocorticoid receptor antagonists, sodium–glucose cotransporter-2 inhibitors, calcium channel blockers, antiplatelets, anticoagulants, antiarrhythmics, and diuretics). These covariates were selected based on their potential association with the clinical profile and risk of patients with TTS and those with HF.

Cox proportional hazards models, before and after PSM, were used to calculate hazard ratios (HRs) and 95% confidence intervals (95% CI) for the risk of adverse events in patients with TTS compared with those with HF. In Cox proportional hazards analyses, cause-specific HRs were estimated, with competing events handled through censoring at the time of their occurrence.

To assess the robustness of the analytic choices and the potential impact of early post-discharge events and follow-up duration, sensitivity analyses were performed by initiating follow-up at the index date (day 0) and by extending follow-up beyond 3 years without an upper time limit.

Aalen–Johansen curves were used to estimate the daily cumulative incidence of the primary outcomes, explicitly accounting for competing risks, with all-cause death treated as a competing event for non-fatal outcomes (MACE and acute heart failure) over follow-up.

The proportional hazards assumption was tested for primary outcomes using Schoenfeld residuals and corresponding Chi-square (χ²) tests. Further details on the performance and interpretation of this test can be found under the ‘Supplementary Methods’ section of the Supplementary material.

As a sensitivity analysis, considering that HF is one of the main clinical manifestations of TTS, we compared patients with both TTS and HF with those with HF alone, using the same matching strategy and analytic framework.

To further explore the heterogeneity of HF, and in light of the predominantly systolic nature of TTS, pre-specified subgroup analyses were performed comparing TTS to both HF with reduced ejection fraction (HFrEF) (ICD-10-CM: I50.2x) and HF with preserved ejection fraction (HFpEF) (ICD-10-CM: I50.3x) cohorts over 3 years, using the same matching strategy.

Additional subgroup analyses were conducted based on age (patients aged ≥65 years and those aged <65 years), and presence or absence of mental health disorders (ICD-10-CM: F01-F99). For each subgroup comparison, separate propensity score matching, and survival analyses were performed using the same matching criteria and statistical methods described for the primary analysis.

The construction of each cohort and the corresponding ICD-10-CM codes for inclusion and exclusion are reported in Supplementary Table S1.

All statistical analyses were performed with the TriNetX analytics platform, which integrates R (v3.2-3) and Python 3.7 libraries (scikit-learn for logistic models; survival for Cox analysis). TriNetX does not perform imputation for missing values. All *P*-values were two-tailed, with statistical significance set at *P* < .05.

## Results

The final cohort included 2240 patients with TTS (mean age 62.6 ± 17.3 years; 73.7% females) and 265,564 patients with HF (mean age 69.3 ± 14.7 years; 45.8% females).

Before PSM, TTS patients were younger and more frequently female, with a lower prevalence of cardiovascular comorbidities, but a higher prevalence of mental health disorders (*Table 1*).

**Table 1:** Baseline characteristics of patients with TTS compared to those with HF

	PrePSM			AfterPSM		
	TTS (*n* = 2240)	HF (*n* = 265 564)	ASD	TTS (*n* = 2238)	HF (*n* = 2238)	ASD
Age at index, mean ± SD	62.6 ± 17.3	69.3 ± 14.7	0.419	62.6 ± 17.3	62.7 ± 17.8	.002
Female, *n* (%)	1651 (73.7)	121 580 (45.8)	0.594	1649 (73.7)	1664 (74.4)	.015
Not Hispanic or Latino, *n* (%)	1821 (81.3)	222 095 (83.6)	0.061	1819 (81.3)	1803 (80.6)	.018
Ischaemic heart disease, *n* (%)	1096 (48.9)	157 689 (59.4)	0.211	1096 (49.0)	1113 (49.7)	.015
Diabetes mellitus, *n* (%)	610 (27.2)	117 234 (44.1)	0.359	610 (27.3)	618 (27.6)	.008
Hypertension, *n* (%)	1431 (63.9)	188 105 (70.8)	0.149	1431 (63.9)	1464 (65.4)	.031
Hyperlipidemia, *n* (%)	1025 (45.8)	151 083 (56.9)	0.224	1024 (45.8)	1036 (46.3)	.011
Atrial fibrillation, *n* (%)	612 (27.3)	107 486 (40.35)	0.281	612 (27.3)	591 (26.4)	.021
Cerebral infarction, *n* (%)	334 (14.9)	30 068 (11.3)	0.106	333 (14.9)	371 (16.6)	.047
Pulmonary embolism, *n* (%)	176 (7.9)	14 820 (5.6)	0.091	176 (7.9)	157 (7.0)	.032
Atherosclerosis of native arteries of the extremities, *n* (%)	78 (3.5)	12 470 (4.6)	0.061	78 (3.5)	82 (3.7)	.010
Obesity, *n* (%)	337 (15.0)	57 963 (21.8)	0.176	337 (15.1)	345 (15.4)	.010
Chronic kidney disease, *n* (%)	452 (20.2)	97 559 (36.7)	0.373	452 (20.2)	453 (20.2)	.001
Mental disorders, *n* (%)	1592 (71.1)	152 067 (57.3)	0.291	1590 (71.0)	1564 (69.9)	.025
Anxiety, *n* (%)	918 (41.0)	67 943 (25.6)	0.331	916 (40.9)	807 (36.1)	.100
Psychoactive substance use, *n* (%)	837 (37.4)	73 233 (27.6)	0.210	836 (37.4)	797 (35.6)	.036
Mood disorders, *n* (%)	810 (36.2)	65 631 (24.7)	0.251	808 (36.1)	752 (33.6)	.053
Mental disorders due to known physiological conditions, *n* (%)	328 (14.6)	34 719 (13.1)	0.045	328 (14.7)	293 (13.1)	.045
Beta blockers, *n* (%)	1763 (78.7)	207 867 (78.3)	0.011	1761 (78.7)	1760 (78.6)	.001
Antiarrhythmics, *n* (%)	1673 (74.7)	179 215 (67.5)	0.159	1671 (74.7)	1659 (74.1)	.012
Diuretics, *n* (%)	1451 (64.8)	209 995 (79.1)	0.322	1451 (64.8)	1448 (64.7)	.003
Calcium channel blockers, *n* (%)	913 (40.8)	133 222 (50.2)	0.190	913 (40.8)	936 (41.8)	.021
ACE inhibitors, *n* (%)	923 (41.2)	104 751 (39.4)	0.036	922 (41.2)	922 (41.2)	<.001
Angiotensin II inhibitor, *n* (%)	499 (22.3)	70 562 (26.6)	0.100	499 (22.3)	451 (20.2)	.052
Anticoagulants, *n* (%)	2019 (90.1)	237 266 (89.3)	0.026	2017 (90.1)	2011 (89.9)	.009
Antiplatelets, *n* (%)	1450 (64.7)	178 900 (67.4)	0.056	1448 (64.7)	1487 (66.4)	.037
ARNI, *n* (%)	40 (1.8)	11 120 (4.2)	0.141	40 (1.8)	37 (1.7)	.010
MRA, *n* (%)	301 (13.4)	45 339 (17.1)	0.101	301 (13.4)	1302 (13.5)	.001
Empagliflozin, *n* (%)	24 (1.1)	4333 (1.6)	0.049	24 (1.1)	22 (1.0)	.009
Dapagliflozin, *n* (%)	10 (0.4)	2395 (0.9)	0.056	10 (0.4)	11 (0.5)	.007
LVEF, mean ± SD	45.1 ± 14.1	49.2 ± 17.1	0.342	45.2 ± 14.1	50.7 ± 17.7	.342

TTS, Takotsubo; HF, heart failure; ACE, angiotensin-converting enzyme; ASD, absolute standardized mean difference.

The number of primary and secondary outcomes, along with corresponding HRs for the comparison before and after PSM, are reported in *Table 2*. Before PSM, patients with TTS were associated with a significantly lower risk of all-cause death (HR 0.657, 95% CI 0.591–0.730), MACE (HR 0.850, 95% CI 0.727–0.994), and acute HF (HR 0.519, 95% CI 0.466–0.577) compared to those with HF. A lower risk was also observed for all secondary outcomes in the TTS cohort, including malignant and ventricular arrhythmias (HR 0.678, 95% CI 0.507–0.905 and HR 0.474, 95% CI 0.356–0.631, respectively), new-onset AF (HR 0.515, 95% CI 0.420–0.632), myocardial infarction (HR 0.720, 95% CI 0.633–0.819), and stroke (HR 0.758, 95% CI 0.594–0.967) (*Table 2*).

**Table 2:** Risks of primary and secondary outcomes in patients with TTS compared to those with HF.

	Pre PSM			After PSM		
	TTS (*n*/*N*)	HF (*n*/*N*)	HR (95% CI)	TTS (*n*/*N*)	HF (*n*/*N*)	HR (95% CI)
All-cause death	348/2288	58 433/269 292	0.657 (0.591, 0.730)	340/2238	396/2239	0.846 (0.732, 1.008)
MACE	159/1680	18 381/174 505	0.850 (0.727, 0.994)	157/1639	125/1378	1.070 (0.846, 1.354)
Acute heart failure	340/2288	68 556/269 292	0.519 (0.466, 0.577)	314/2049	479/2049	0.622 (0.539, 0.717)
Malignant arrhythmias	46/2063	7944/258 009	0.678 (0.507, 0.905)	46/2021	59/2129	0.656 (0.571, 0.754)
Ventricular arrhythmias	47/2054	10 968/243 543	0.474 (0.356, 0.631)	47/2010	72/2009	0.637 (0.441, 0.919)
New onset AF	93/1618	16 034/153 847	0.515 (0.420, 0.632)	93/1584	136/1594	0.672 (0.517, 0.875)
Myocardial infarction	235/2288	35 692/269 292	0.720 (0.633, 0.819)	233/2238	276/2238	0.818 (0.687, 0.974)
Stroke	65/1893	9847/234 167	0.758 (0.594, 0.967)	65/1848	66/1839	0.965 (0.685, 1.360)

TTS, Takotsubo; HF, Heart failure; MACE, Major Adverse Cardiovascular Events; AF, Atrial fibrillation.

*n*/*N* indicates number of events (*n*) over the total number of patients included in the analysis (*N*).

**Figure 1:**
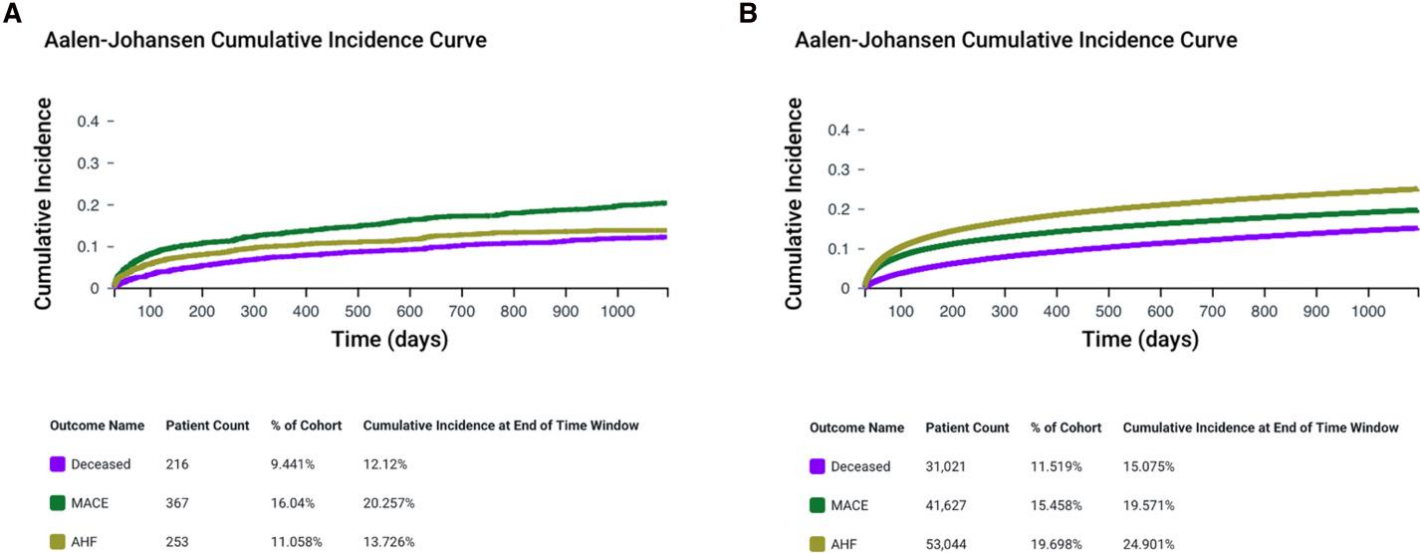
Aalen–Johansen curves before propensity score matching for the cumulative incidence of primary outcomes in patients with TTS (Panel *A*) and HF (Panel *B*). TTS indicates Takotsubo; HF indicates heart failure; MACE indicates major adverse cardiovascular events; AHF indicates acute heart failure

Aalen–Johansen curves before PSM in TTS and HF patients are reported in *Figure 1*. The 3-year cumulative incidences for our primary outcomes were as follows: 12.12% and 15.1% for all-cause death; 20% and 20% for MACE; 14% and 25% for acute HF, respectively (*Figure 1*, Panels *A* and *B*).

After PSM, baseline characteristics, including demographics, cardiovascular comorbidities, and medication use, were well balanced across groups (ASD <0.1) (*Table 1*). Compared with HF patients, those with TTS showed no statistically significant difference in the risk of all-cause death (HR 0.846, 95% CI 0.732–1.008) and MACE (HR 1.070, 95% CI 0.846–1.354) (*Table 2*). TTS was still associated with a significantly lower risk of acute HF (HR 0.622, 95% CI 0.539–0.717) (*Table 2*). Among secondary outcomes, lower risks were observed in the TTS cohort for malignant and ventricular arrhythmias (HR 0.656, 95% CI 0.571–0.754 and HR 0.637, 95% CI 0.441–0.919, respectively), new-onset AF (HR 0.672, 95% CI 0.517–0.875), and myocardial infarction (HR 0.818, 95% CI 0.687–0.974), with no significant difference in stroke (HR 0.965, 95% CI 0.685–1.360) (*Table 2*).

Sensitivity analyses starting follow-up at day 0 and extending follow-up beyond 3 years yielded results consistent with the primary analysis, with stable effect estimates over time (Supplementary Table S3).

When testing the proportional hazards assumption for the 3-year risk of primary outcomes in TTS patients compared with HF patients after PSM, no violations were observed for all the three outcomes (all-cause death: χ² = 0.005, *P* = .946; MACE: χ² = 0.084, *P* = .772; acute HF: χ² = 0.005, *P* = .942) (Supplementary Figure S2).

### Sensitivity analyses

#### TTS with HF vs HF without TTS

Restricting the analysis only to TTS patients with HF, 1103 matched pairs were included after PSM. In this sensitivity analysis, the prognostic gap with HF was attenuated, with TTS and HF patients showing lower risks of acute HF (HR 0.733, 95% CI 0.612–0.877), myocardial infarction (HR 0.636, 95% CI 0.492–0.821), and new-onset AF (HR 0.691, 95% CI 0.487–0.981), but no statistically significant differences for other outcomes (Supplementary Table S4).

#### TTS vs HFrEF and HFpEF: risk profile by HF phenotype

In analyses stratified by HF phenotype, patients with TTS exhibited a more favourable long-term risk profile compared to those with HFrEF, especially for arrhythmic and decompensation-related events.

Compared with HFrEF, TTS was associated with a significantly lower risk of acute HF (HR 0.467, 95% CI 0.409–0.533), ventricular arrhythmias (HR 0.490, 95% CI 0.344–0.697), new-onset AF (HR 0.681, 95% CI 0.523–0.888), and myocardial infarction (HR 0.634, 95% CI 0.537–0.749). No statistically significant differences were found for all-cause death, MACE, malignant arrhythmias, and stroke (Supplementary Table S5).

In the comparison with HFpEF, the same directional pattern emerged but was less marked. Significant risk reductions were limited to acute HF (HR 0.629, 95% CI 0.548–0.721) and new-onset AF (HR 0.700, 95% CI 0.536–0.913), with no statistically significant differences for the other outcomes (Supplementary Table S5).

#### TTS vs HF stratified by age: trends in younger and older adults

Age-stratified analyses confirmed similar trends across groups.

**Figure 2:**
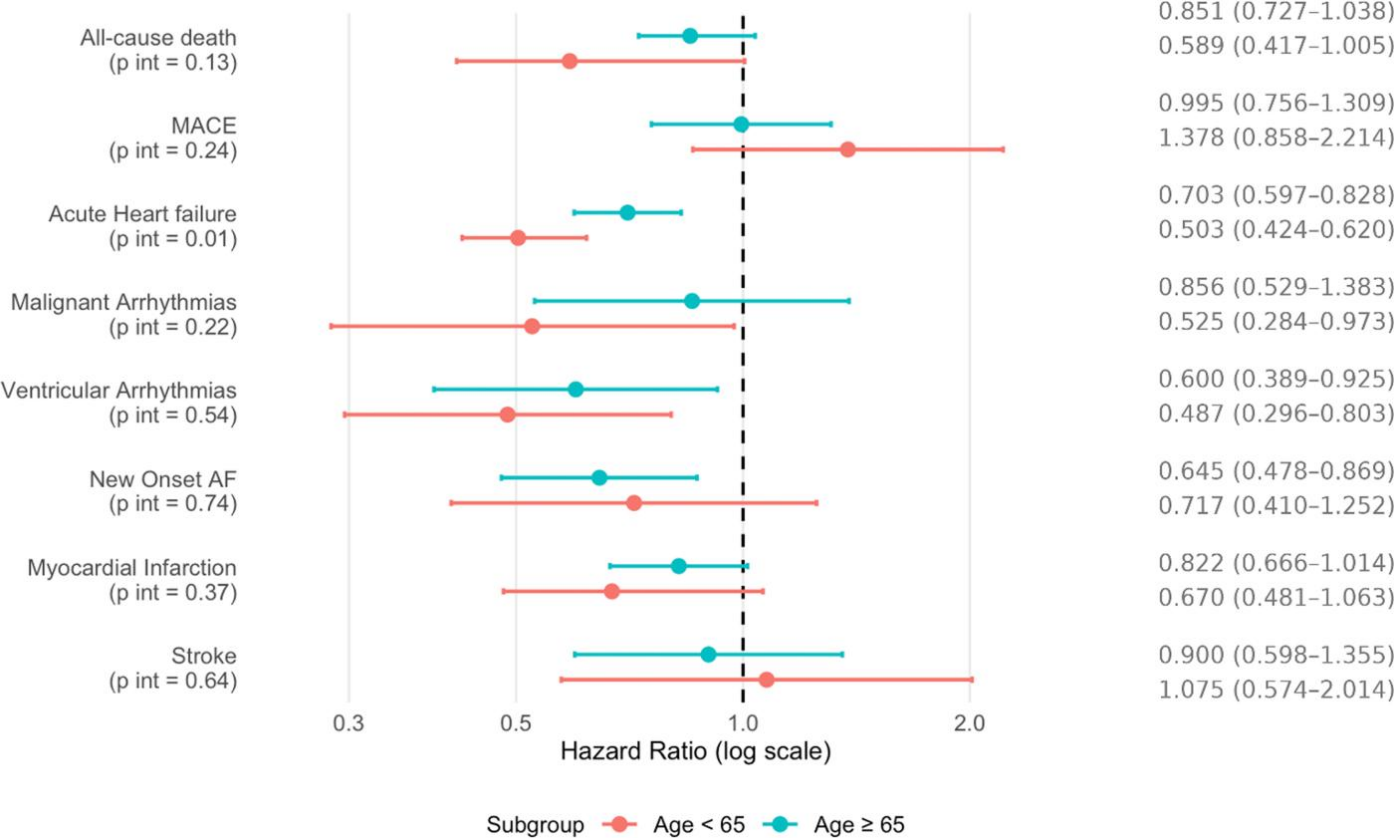
Risks of primary and secondary outcomes in patients with TTS compared to those with HF in clinically relevant subgroups of patients (age ≥65 years, <65 years). HRs with 95% CIs shown in parentheses are shown for each outcome. *P* values for interaction between subgroups are reported in parentheses beneath each outcome label. TTS indicates Takotsubo; HF, heart failure; MACE, major adverse cardiovascular events; AF, atrial fibrillation

Among patients aged ≥65 years, the differences between TTS and HF were narrower, with statistically significant reductions limited to acute HF (HR 0.703, 95% CI 0.597–0.828), ventricular arrhythmias (HR 0.600, 95% CI 0.389–0.925), and new-onset AF (HR 0.645, 95% CI 0.478–0.869). No significant differences were observed for all-cause death, MACE, stroke, malignant arrhythmias, and myocardial infarction (*Figure 2*; Supplementary Table S6).

Among patients aged <65 years, differences were more pronounced, with TTS associated with significantly lower risks of acute HF (HR 0.553, 95% CI 0.424–0.720), malignant and ventricular arrhythmias (HR 0.553, 95% CI 0.424–0.720 and HR 0.525, 95% CI 0.284–0.973, respectively), while no significant differences were found for the other outcomes (*Figure 2*; Supplementary Table S6).

When comparing TTS to specific HF phenotypes within each age group, the observed differences were broadly consistent with the main analysis.

Among older patients (≥65 years), TTS was associated with a significantly lower risk of acute HF compared to both HFrEF and HFpEF, while a lower risk of new-onset AF was observed only in the comparison with HFrEF; no statistically significant differences were observed for all-cause death in this age group (Supplementary Tables S7 and S8).

Among younger patients (<65 years), TTS was associated with lower risks of acute HF compared with both HFrEF and HFpEF, with reductions in ventricular arrhythmias that were more pronounced in comparisons with HFrEF. In this age group, TTS was also associated with significantly lower risks of myocardial infarction and all-cause death compared to both HF phenotypes, whereas no significant differences were observed for stroke (Supplementary Tables S7 and S8).

#### TTS vs HF in patients with and without mental health disorders

**Figure 3:**
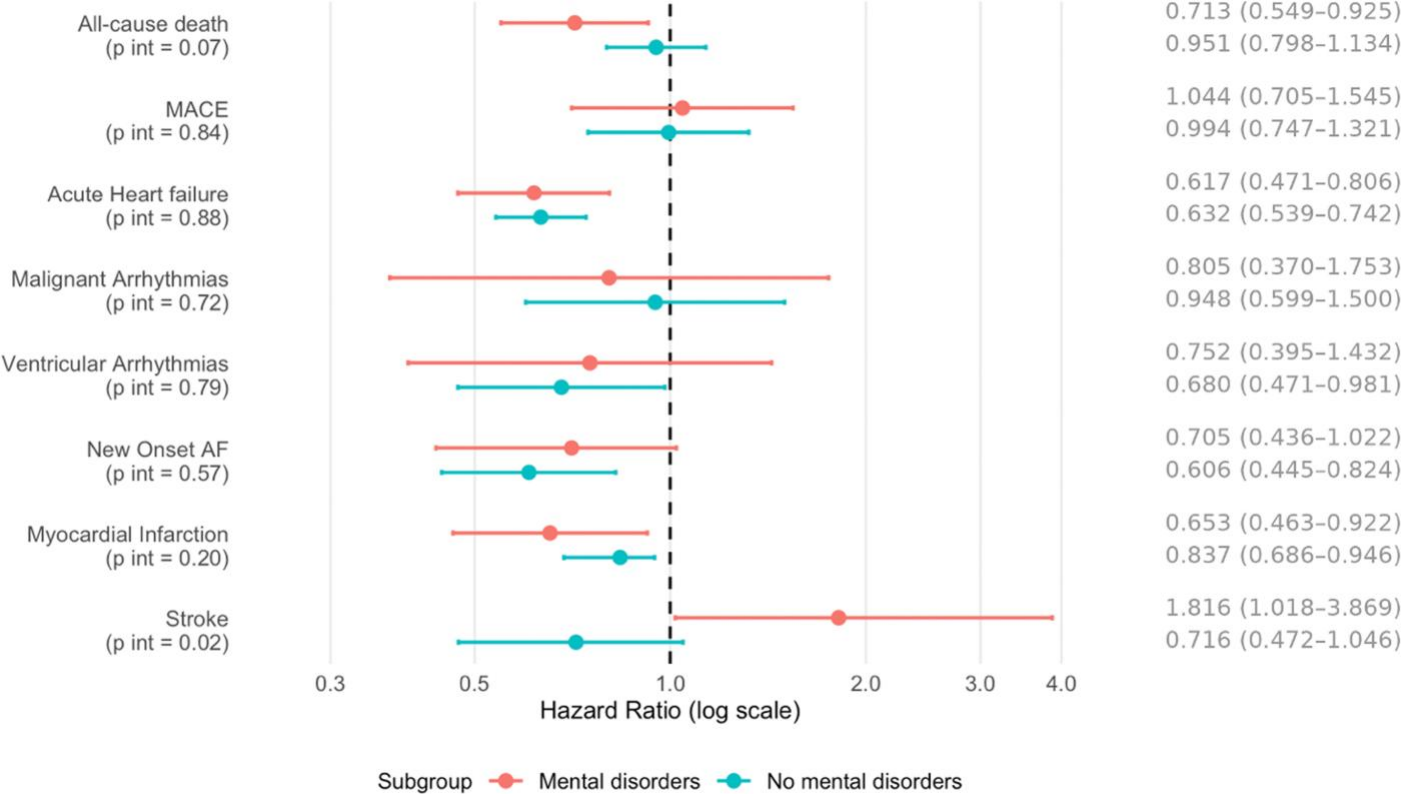
Risks of primary and secondary outcomes in patients with TTS compared with those whit HF in clinically relevant subgroups of patients (presence of mental health disorders, mental disorder; absence of mental health disorders, no mental disorder). HRs with 95% confidence intervals (CIs) shown in parentheses are shown for each outcome. *P* values for interaction between subgroups are reported in parentheses beneath each outcome label. TTS, Takotsubo; HF, heart failure; MACE, major adverse cardiovascular events; AF, atrial fibrillation

In the subgroup of patients with documented mental health disorders, TTS was associated with a significantly lower risk of all-cause death (HR 0.713, 95% CI 0.549–0.925), acute HF (HR 0.617, 95% CI 0.471–0.806), and myocardial infarction (HR 0.653, 95% CI 0.463–0.922), but with a notably increased risk of stroke (HR 1.816, 95% CI 1.018–3.869). No significant differences were found for the other outcomes (*Figure 3*; Supplementary Table S9). Among patients without documented mental health disorders, the prognostic gap between TTS and HF was less pronounced. In this subgroup, TTS remained associated with lower risks of acute HF (HR 0.632, 95% CI 0.535–0.747), ventricular arrhythmias (HR 0.680, 95% CI 0.471–0.981), new-onset AF (HR 0.606, 95% CI 0.445–0.824), and myocardial infarction (HR 0.837, 95% CI 0.472–0.946), with no significant differences observed for all-cause death, MACE, malignant arrhythmias, or stroke (*Figure 3*; Supplementary Table S9).

## Discussion

In our study, our principal findings are as follows: (i) patients with TTS had a lower 3-year risk of acute HF, ventricular arrhythmias, myocardial infarction, and new-onset AF compared to those with HF, although no difference was found for the risk of all-cause death, MACE, and stroke; (ii) the lower risk of adverse events was more pronounced when compared to patients with HFrEF, while it was similar when compared to those with HFpEF; (iii) the risk of acute HF associated with TTS was lower in younger patients and in those with mental disorders, compared with older patients and those without mental disorders, respectively. Conversely, the risk of stroke in patients with TTS was higher in younger patients and in those with mental disorders than in older patients and those without mental disorders.

The lower risk of cardiovascular events observed in patients with TTS compared to those with HF may be explained by the fact that, although both conditions can present with acute haemodynamic compromise, TTS typically follows a reversible course, with recovery of left ventricular function and a lower long-term burden of chronic cardiovascular morbidity.^13^

While no direct comparative studies are currently available, valuable insights into the long-term cardiovascular risk associated with TTS and HF can be drawn from registry-based cohorts and randomized clinical trials, suggesting a diverging clinical pattern: in TTS, cardiovascular risk appears to decrease over time, while in HF it tends to persist or worsen progressively.

This time-dependent trend is well illustrated in the InterTAK Registry, which included over 1700 patients with TTS and reported a 10-year cumulative incidence of MACE—including cardiac death and stroke—of approximately 15%, with the majority of events occurring in the initial years of follow-up.^14,15^ This trend of long-term risk attenuation is further supported by findings from the GEIST registry, a European multicenter cohort including patients with emotionally triggered TTS, where the 5-year incidence of cardiovascular events and rehospitalizations was reported to be below 10%.^16^

This trajectory stands in stark contrast to what is observed in HF, where chronic myocardial alterations likely underlie a persistently high—and progressively worsening—cardiovascular risk.

Consistently, data from major clinical trials have shown that, even under guideline-directed medical therapy, the prognosis of HF remains poor over time, regardless of ejection fraction.^17,18^ In PARADIGM-HF, the annual rate of cardiovascular death or HF hospitalization was approximately 13%^19^ and similar event rates, ranging from 10% to 15% per year, have been reported across landmark trials in HF, including DAPA-HF and EMPHASIS-HF for reduced ejection fraction,^20,21^ as well as EMPEROR-Preserved and TOPCAT in patients with preserved ejection fraction.^22,23^

Although no direct comparisons of all-cause mortality between TTS and HF are available, a recent meta-analysis in TTS reported an annual all-cause mortality rate of approximately 3.5%.^24^ While this estimate includes both cardiovascular and non-cardiovascular causes, its substantially lower value compared with cardiovascular mortality rates observed in HF trials strongly suggest that cardiovascular mortality is also reduced in patients with TTS.

As demonstrated in our analyses, comparing disease entities in isolation may obscure clinically relevant heterogeneity: when focusing specifically on patients with TTS who experienced HF during the index hospitalization, outcome differences were substantially reduced, with a risk profile closer to that of HF. This observation aligns with prior data from the RETAKO registry, where TTS patients with delayed recovery of left ventricular function or in-hospital acute heart failure showed significantly higher long-term mortality.^25^ However, given the predominantly systolic nature of TTS, findings from pooled HF analyses warrant cautious interpretation, as risk profiles further diverge when examined across HF phenotypes. Indeed, while TTS generally demonstrates a lower risk of adverse events compared to HF overall, this advantage becomes even more nuanced when outcomes were examined within specific HF phenotypes.

In our analysis, the risk gap between TTS and HFrEF appeared wider than that observed between TTS and HFpEF. This observation aligns with established differences in the natural history of heart failure phenotypes. Large-scale registries and meta-analyses have consistently demonstrated that patients with HFrEF experience higher rates of adverse cardiovascular events and mortality compared to those with HFpEF. For instance, the MAGGIC meta-analysis, encompassing data from over 30 observational studies, reported a 32% lower adjusted risk of 3-year mortality in HFpEF patients compared with HFrEF counterparts.^26^ Similarly, data from the Swedish Heart Failure Registry indicated that the risk of 1-year mortality was higher in HFrEF compared with HFpEF.^27,28^

These differences are likely attributable to variations in myocardial remodelling, neurohormonal activation, and the prevalence of ischaemic heart disease between the two phenotypes.^29,30^ HFrEF is predominantly characterized by systolic dysfunction resulting from myocardial loss or injury, maladaptive eccentric remodelling, and robust activation of neurohormonal pathways. In contrast, HFpEF is largely driven by diastolic dysfunction, systemic inflammation, and microvascular endothelial impairment, leading to concentric hypertrophy, increased myocardial stiffness, and impaired relaxation.

This differential risk profile may help explain why the long-term outcomes of TTS and HFpEF appear numerically closer than those observed between TTS and HFrEF. However, among the outcomes assessed, one notable area of divergence was the arrhythmic profile—specifically, the incidence of new-onset atrial fibrillation, which was significantly higher in HFpEF compared with TTS, despite otherwise comparable risks.

This finding may reflect underlying differences in atrial structure and function. In HFpEF, left atrial remodelling and elevated filling pressures contribute to a substrate highly prone to atrial fibrillation, which in turn exacerbates diastolic dysfunction and predisposes to further haemodynamic instability.^31^ This bidirectional relationship has led to the concept of ‘atrial cardiomyopathy,’ wherein structural and functional atrial changes actively drive the development and progression of both HFpEF and AF.^32,33^ Notably, AF is not merely a consequence but also a contributor to HFpEF pathogenesis—an association acknowledged in diagnostic frameworks such as the HFA-PEFF score, where its presence carries significant diagnostic weight.^34^ In contrast, in TTS, atrial arrhythmias may occur in the acute setting due to transient autonomic dysregulation or catecholaminergic surge, but sustained atrial remodelling is generally absent. This relative structural sparing of the atria may help explain the lower incidence of new-onset AF observed in TTS over long-term follow-up.^35^

Beyond the intrinsic features of each syndrome, patient characteristics may further influence the long-term trajectory of both TTS and HF. Among these, age represents a key modulatory factor, potentially shaping clinical expression, vulnerability to complications, and overall prognosis.

Indeed, stratification by age revealed important differences in the comparative outcomes between TTS and HF. When comparing patients aged ≥65 years, the lower risk of adverse events of TTS appeared less pronounced, whereas in younger individuals (<65 years), outcome differences widened considerably.

According to data from a large international registry, long-term mortality in TTS shows a clear age-related gradient, increasing from 5.6% in patients under 45 years up to 22.3% in those aged 75 and older.^8,36^ Younger patients with TTS typically present with the classic stress-induced form of the syndrome, in the absence of significant structural heart disease or comorbidities, which likely explains their rapid recovery and favourable prognosis. Conversely, older individuals tend to carry a higher burden of cardiovascular risk factors, subclinical myocardial fibrosis, and atrial remodelling, all of which may blunt the transient nature of TTS and predispose to adverse outcomes.^37,38^

While advanced age in HF patients is commonly associated with poorer outcomes due to the accumulation of comorbidities, it’s crucial to recognize that younger patients are not inherently protected from adverse prognoses. In fact, certain aetiologies prevalent among younger individuals can lead to outcomes that are as severe, if not worse, than those observed in older populations.^39^ A comprehensive analysis from the Swedish Heart Failure Registry, encompassing over 60 000 patients, revealed that individuals under 55 years of age exhibited a 1-year all-cause mortality rate of 4.2%, compared with 0.3% in age-matched controls. Notably, the relative mortality risk was highest among the youngest cohort, with those aged 18–34 years experiencing a hazard ratio of 38.3 when compared to controls. Furthermore, at the age of 20, the estimated life-years lost due to HF was up to 36 years for 50% of patients, underscoring the profound impact of HF in younger populations.^39^

These findings illustrate that both age and disease substrate must be considered in prognostication. While advanced age often implies greater comorbidity and frailty,^40^ younger age does not exclude severe or rapidly progressive disease. Ultimately, a comprehensive, multidimensional assessment is essential—one that goes beyond the diagnostic label to incorporate age, aetiology, comorbidities, and psychosocial context. This approach is especially important when addressing populations with additional vulnerability factors, such as those with mental health disorders.

In our study, patients with mental health disorders exhibited a reduced risk of adverse events in the context of TTS compared with HF, with the notable exception of an increased risk of stroke.

Mental health disorders—particularly depression and anxiety—are highly prevalent in individuals with cardiovascular disease and are well-established determinants of worse clinical outcomes.^41^ They influence prognosis both directly, through autonomic imbalance, inflammation, and endothelial dysfunction, and indirectly, by impairing adherence to therapy and limiting engagement in healthy behaviours.

In TTS, however, these disorders play a particularly complex role. Beyond being common comorbidities, they often act as the very triggers of the syndrome.^8^ Intriguingly, emotional stressors as precipitants are associated with more favourable in-hospital and long-term outcomes compared with physical triggers or no identifiable cause^42^—an observation that may partially support the overall prognostic advantage of TTS over HF in this subgroup. However, the observed association between mental health disorders and stroke risk in TTS should be interpreted cautiously, as unmeasured factors—including antithrombotic therapy, atrial substrate, left ventricular thrombus, and the use of psychotropic medications—may have contributed to residual confounding.

This same finding may nonetheless offer pathophysiological insight. While emotional triggers may be linked to better general outcomes, the physiological response they provoke—particularly sympathetic overactivation—may exacerbate the inherent embolic risk of TTS. Transient systolic dysfunction and apical ballooning create a prothrombotic state, which, compounded by autonomic dysregulation and increased platelet reactivity, may amplify the risk of stroke.^8,43^

Together, these results underscore the dual nature of mental health disorders in TTS—associated with improved overall prognosis, yet potentially contributing to specific adverse events such as stroke. Recognizing this complexity is key to optimizing management strategies and ensuring appropriate cerebrovascular risk assessment in this vulnerable population.

### Limitations

Several limitations should be considered when interpreting the findings of this study. First, its retrospective observational design inherently limits causal inference and may be subject to selection bias and residual confounding. Second, the identification of exposures and outcomes relied on administrative codes (ICD-10-CM and CPT), which lack clinical granularity and do not capture key prognostic features such as the severity of acute heart failure at presentation, longitudinal recovery of left ventricular function, objective frailty measures, or socioeconomic status. Although PSM balanced an extensive set of clinical variables—including baseline LVEF and contemporary guideline-directed medical therapy—residual confounding related to disease severity, treatment adherence, and post-discharge management cannot be excluded. Third, the exclusion of patients with prior events in the baseline period—implemented to avoid confounding—may have led to the omission of individuals at highest risk, potentially underestimating the true event burden and affecting generalizability.

Fourth, the classification of mental health disorders did not account for severity, chronicity, or treatment status, which may substantially influence cardiovascular risk and prognosis. Fifth, our analysis did not evaluate the potential role of concomitant pharmacotherapies, including psychotropic agents or antithrombotic regimens, which may impact outcomes such as stroke.

Sixth, outcomes occurring outside of the contributing healthcare organizations may have been missed, leading to possible outcome misclassification. Seventh, subgroup analyses by age, HF phenotype, and psychiatric comorbidities were exploratory and not powered to detect small differences; thus, findings should be interpreted cautiously. Finally, data on race, ethnicity, and socioeconomic status were incompletely captured and not adjusted for in all models, limiting our ability to assess disparities in care or outcomes. This may be important given the reported ethnic differences in cardiovascular disease related outcomes, such as stroke and bleeding.^44,45^

## Conclusion

TTS is associated with a lower risk of adverse events compared with HF—a difference that was more pronounced when compared with HFrEF, but less evident when compared with HFpEF.

These findings reinforce the concept of TTS as a distinct clinical entity with a milder long-term cardiovascular trajectory, highlighting the need for further studies into its pathogenesis, and the complex role of mental health in shaping clinical outcomes.

## Supplementary Material

xvag065_Supplementary_Data

## Declarations

### Data Availability

Data used in this research project will be available upon a reasonable request from the corresponding authors.
