# Supplementary material for: Long-term prognosis in Takotsubo syndrome compared to heart failure: observations from a global federated research network
Source: ESC Heart Fail. 2026 Feb 27;13(2):xvag065. doi: 10.1093/eschf/xvag065 (PMC13012821; doi:10.1093/eschf/xvag065)

**Long-term prognosis in Takotsubo Syndrome compared to Heart Failure: Observations**

**from a global federated research network.**

Enrico Tartaglia, M. Alobaida, Tommaso Bucci, Michele Rossi, Amir Askarinejad, Ho Man Lam, M. Kaskal, Andrea Galeazzo Rigutini, Giuseppe Boriani, Gregory Y. H. Lip

Supplementary material

***Supplementary Methods***

TriNetX Database

The TriNetX data are collected from member healthcare organizations (HCO) and originates from their primary electronic health records (EHR) system. A typical HCO is a large academic health center with data coming from majority of its affiliates. A single HCO frequently has more than one facility, including main and satellite hospitals. The data are stored on the TriNetX database via a physical server at the institution’s data centre or a virtual hosted appliance. The TriNetX platform comprises of a series of these appliances connected into a federated network. This network can broadcast queries to each appliance. Results are subsequently collected and aggregated. Once the data are sent to the network, it is mapped to a standard and controlled set of clinical terminologies and undergoes a data quality assessment including ‘data cleaning’ that rejects records which do not meet the TriNetX quality standards. The TriNetX database performs internal and extensive data quality assessment with every refresh based on conformance, completeness, and plausibility (http://doi.org/10.13063/2327-9214.1244). HIPAA (Health Insurance Portability and Accountability Act) compliance of the clinical patient data is achieved using deidentification. Available data types within the network include demographics, diagnoses (represented by ICD-10-CM codes), procedures (coded in ICD-10-PCS or CPT), and measurements (coded to LOINC). While extensive information is provided about patients’ diagnoses and procedures, other variables (such as socioeconomic and lifetime factors are not comprehensively represented). The advantage of EHR data over insurance claim data is that both insured and uninsured patients are included. An advantage of EHR data over survey data is that the former represents the diagnostic rates in the population presenting to healthcare facilities. This provides an accurate account of the burden of specific diagnoses on healthcare systems. One primary limitation of relying on diagnoses is that they do not account for undiagnosed patients who might have a condition but have not yet received medical support. Another general limitation of EHR data is that a patient may be seen in different HCO for different components of their care. If one HCO is not part of the federated network, then part of their medical records may not be available. Using a network of healthcare organizations, rather than a single site, limits this possibility but does not fully remove it.

Propensity Score Matched Analyses were performed using logistic regression [Logistic Regression from the scikit-learn package in Python (version 3.7)]. TriNetX performed a 1:1 greedy nearest neighbor matching model with a caliper of 0.1 pooled standard deviations. To eliminate bias resulting from nearest neighbor algorithms, the rows were randomized. Any baseline characteristic with a standardized mean difference between cohorts lower than 0.1 was deemed well matched. (<https://www.tandfonline.com/doi/full/10.1080/00273171.2011.568786>).

Assessment of the Proportional Hazards Assumption

To evaluate whether the proportional hazards assumption was satisfied in the Cox regression models, we conducted a Chi-square (χ²) test based on Schoenfeld residuals. These tests examine whether the relationship between the associated variables and the hazard function remains stable over time. The null hypothesis posits that the effect of OAC discontinuation on the hazards of primary outcomes is constant throughout the study period. The χ² statistic measures the discrepancy between the observed and expected Schoenfeld residuals. A higher χ² value indicates a greater divergence from the expected values, suggesting a potential violation of the proportional hazards assumption. Conversely, a lower χ² value implies that the observed residuals closely align with the expected values, supporting the assumption. The p-value, derived from the χ² statistic, reflects the likelihood of observing these deviations under the null hypothesis. A p-value greater than 0.05 suggests that the deviations are likely due to random variation, indicating that the proportional hazards assumption holds. In contrast, a p-value less than 0.05 implies that the observed deviations are unlikely to be random, indicating a violation of the proportional hazards assumption.

**Supplementary Table S1**. ICD-10-CM codes for inclusion and exclusion criteria in patients with Takotsubo or Heart Failure. In subgroup analyses stratified by age (≥65 and <65 years), the only criterion that differs from the main cohort definitions is the age threshold. TTS indicates Takotsubo Syndrome; HF indicates Heart Failure; HFrEF indicates Heart Failure with reduced Ejection Fraction; HFpEF indicates Heart Failure with preserved Ejection Fraction; ICD-10 indicates International Classification of Diseases, 10th Revision (diagnosis codes); CPT indicates Current Procedural Terminology (procedure codes).

| **Cohort** | **Comparison** | **Age (inclusion)** | **Included Diagnoses** | **Excluded Diagnoses** | **Time Period** | **Discharge Required** | **Temporal Relationship to Index Event** |
| --- | --- | --- | --- | --- | --- | --- | --- |
| *TTS* | TTS vs HF | ≥18 (subgroup: ≥65 or <65) | Takotsubo syndrome (I51.81) | Acute myocardial infarction (I21) | 1 Mar 2018 - 1 Mar 2022 | Yes (CPT: 1013682) | TTS diagnosis within 1 year prior to or at the time of discharge |
| *HF* | TTS vs HF | ≥18 (subgroup: ≥65 or <65) | Heart failure (I50) | Takotsubo syndrome (I51.81) | 1 Mar 2018 - 1 Mar 2022 | Yes (CPT: 1013682) | HF diagnosis within 1 year prior to or at the time of discharge |
| *TTS* | TTS vs HFrEF | ≥18 (subgroup: ≥65 or <65) | Takotsubo syndrome (I51.81) | Acute myocardial infarction (I21) | 1 Mar 2018 - 1 Mar 2022 | Yes (CPT: 1013682) | TTS diagnosis within 1 year prior to or at the time of discharge |
| *HFrEF* | TTS vs HFrEF | ≥18 (subgroup: ≥65 or <65) | Heart failure (I50) AND Heart failure (I50).2 | Takotsubo syndrome (I51.81) OR Heart failure (I50).3 | 1 Mar 2018 - 1 Mar 2022 | Yes (CPT: 1013682) | HFrEF diagnosis within 1 year prior to or at the time of discharge |
| *TTS* | TTS vs HFpEF | ≥18 (subgroup: ≥65 or <65) | Takotsubo syndrome (I51.81) | Acute myocardial infarction (I21) | 1 Mar 2018 - 1 Mar 2022 | Yes (CPT: 1013682) | TTS diagnosis within 1 year prior to or at the time of discharge |
| *HFpEF* | TTS vs HFpEF | ≥18 (subgroup: ≥65 or <65) | Heart failure (I50) AND Heart failure (I50).3 | Takotsubo syndrome (I51.81) OR Heart failure (I50).2 | 1 Mar 2018 - 1 Mar 2022 | Yes (CPT: 1013682) | HFpEF diagnosis within 1 year prior to or at the time of discharge |
| *TTS (mental)* | TTS vs HF (mental) | ≥18 | Takotsubo syndrome (I51.81) AND Mental, Behavioral and Neurodevelopmental disorders (F01-F99) | Acute myocardial infarction (I21) | 1 Mar 2018 - 1 Mar 2022 | Yes (CPT: 1013682) | TTS diagnosis within 1 year prior to or at the time of discharge |
| *HF (mental)* | TTS vs HF (mental) | ≥18 | Heart failure (I50) AND Mental, Behavioral and Neurodevelopmental disorders (F01-F99) | Takotsubo syndrome (I51.81) | 1 Mar 2018 - 1 Mar 2022 | Yes (CPT: 1013682) | HF diagnosis within 1 year prior to or at the time of discharge |
| *TTS (no mental)* | TTS vs HF (no mental) | ≥18 | Takotsubo syndrome (I51.81) | Acute myocardial infarction (I21) OR Mental, Behavioral and Neurodevelopmental disorders (F01-F99) | 1 Mar 2018 - 1 Mar 2022 | Yes (CPT: 1013682) | TTS diagnosis within 1 year prior to or at the time of discharge |
| *HF (no mental)* | TTS vs HF (no mental) | ≥18 | Heart failure (I50) | Takotsubo syndrome (I51.81) OR Mental, Behavioral and Neurodevelopmental disorders (F01-F99) | 1 Mar 2018 - 1 Mar 2022 | Yes (CPT: 1013682) | HF diagnosis within 1 year prior to or at the time of discharge |

**Supplementary Table S2.** ICD-10-CM codes for the 1-year risk of all-cause death, thrombotic events, and bleeding.

| **Outcome** | **ICD-10-CM Description** |
| --- | --- |
| *Death* | Deceased (demographic status, no ICD-10-CM code) |
| *MACE* | Acute myocardial infarction (I21) OR cerebral infarction (I63) |
| *AHF* | Acute systolic (I50.21) OR acute on chronic systolic (I50.23) OR acute diastolic (I50.31) OR acute on chronic diastolic (I50.33) OR acute combined systolic and diastolic (I50.41) OR acute on chronic combined systolic and diastolic (I50.43) |
| ***Malignant*** *arrhythmias* | Ventricular fibrillation and flutter (I49.0) OR cardiac arrest (I46) |
| ***Ventricular arrhythmias*** | **Ventricular tachycardia (I47.2)** |
| *New-onset AF* | Atrial fibrillation and flutter (I48) |
| *MI* | Acute myocardial infarction (I21) |
| *Stroke* | Cerebral infarction (I63) |

**Supplementary Figure S2.** Risks of primary outcomes in patients with TTS compared to those whit HF. TTS indicates Takotsubo; HF indicates Heart failure; MACE indicates Major Adverse Cardiovascular Events; AF indicates Atrial fibrillation; CI indicates Confidence Intervals; HR, Hazard Ratio. A high χ2 suggests a greater deviation from the expected values, indicating a potential violation of the proportional hazard assumption. Conversely, a small χ2 value indicates that the observed residuals closely match the expected values.


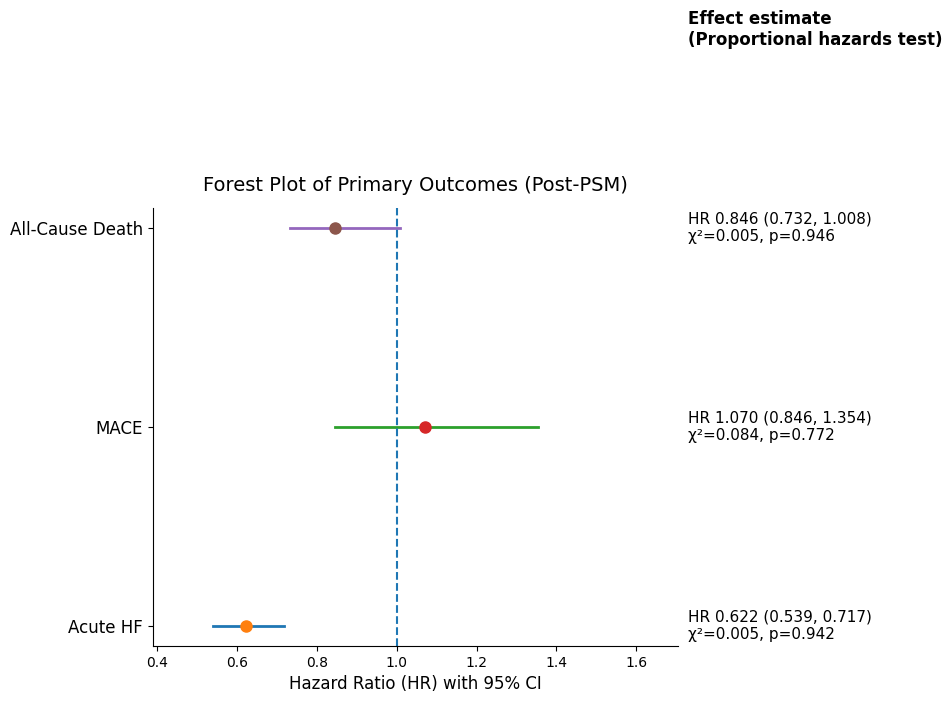


**Supplementary Table S3.**Risks of primary and secondary outcomes in patients with Takotsubo syndrome (TTS) compared with heart failure (HF) in sensitivity analyses starting follow-up at day 0 (0) and extending follow-up beyond three years (LT). Hazard ratios (HRs) are reported with 95% confidence intervals (CIs) shown in parentheses. TTS indicates Takotsubo syndrome; HF, heart failure; MACE, major adverse cardiovascular events; AF, atrial fibrillation. *n/N* indicates the number of events (*n*) over the total number of patients included in the analysis (*N*).

|  | **TTS 0**  **(n/N)** | **HF 0**  **(n/N)** | **HR (95% CI)** | **TTS LT**  **(n/N)** | **HF LT**  **(n/N)** | **HR (95% CI)** |
| --- | --- | --- | --- | --- | --- | --- |
| **All-cause death** | **477/2238** | **513/2238** | **0.923 (0.814, 1.045)** | **495/2238** | **548/2238** | **0.884 (0.783–0.999)** |
| **MACE** | **218/1700** | **174/1427** | **1.066 (0.874, 1.302)** | **211/1639** | **185/1378** | **0.971 (0.797, 1.183)** |
| **Acute Heart failure** | **492/2238** | **671/2238** | **0.693 (0.616, 0.778)** | **107/1352** | **164/1233** | **0.649 (0.571, 0.738)** |
| **Malignant Arrhythmias** | **63/2038** | **69/2139** | **0.945 (0.672, 1.330)** | **69/2021** | **87/2129** | **0.820 (0.598, 1.125)** |
| **Ventricular Arrhythmias** | **64/2027** | **95/2032** | **0.662 (0.482, 0.909)** | **75/2010** | **100/2009** | **0.724 (0.537, 0.977)** |
| **New Onset AF** | **125/1616** | **185/1643** | **0.671 (0.535, 0.842)** | **137/1584** | **200/1594** | **0.658 (0.529, 0.818)** |
| **Myocardial Infarction** | **278/2238** | **371/2238** | **0.716 (0.613, 0.837)** | **294/2238** | **344/2238** | **0.826 (0.707, 0.965)** |
| **Stroke** | **101/1884** | **77/1850** | **1.290 (0.959, 1.736)** | **97/1848** | **95/1839** | **0.996 (0.751, 1.322)** |

**Supplementary Table S4.** Risks of primary and secondary outcomes in patients with TTS+HF compared to those whit HF. HRs are reported with 95% confidence intervals shown in parentheses. TTS indicates Takotsubo; HF indicates Heart failure; MACE indicates Major Adverse Cardiovascular Events; AF indicates Atrial fibrillation. n/N indicates number of events (n) over the total number of patients included in the analysis (N).

|  | **TTS+HF**  **(n/N)** | **HF**  **(n/N)** | **HR (95% CI)** |
| --- | --- | --- | --- |
| **All-cause death** | **162/1103** | **197/1103** | **0.848 (0.689–1.044)** |
| **MACE** | **75/850** | **81/710** | **0.794 (0.580–1.087)** |
| **Acute Heart failure** | **26/391** | **60/571** | **0.733 (0.612–0.877)** |
| **Malignant arrhythmias** | **25/993** | **31/1059** | **0.881 (0.520–1.493)** |
| **Ventricular arrhythmias** | **29/976** | **48/998** | **0.639 (0.403–1.013)** |
| **New-onset AF** | **53/764** | **77/756** | **0.691 (0.487–0.981)** |
| **Myocardial Infarction** | **96/1103** | **151/1103** | **0.636 (0.492–0.821)** |
| **Stroke** | **35/927** | **30/941** | **1.206 (0.740–1.964)** |

**Supplementary Table S5.** Risks of primary and secondary outcomes in patients with TTS compared to those whit HFrEF and HFpEF. TTS indicates Takotsubo; HFrEF indicates heart failure with reduced ejection fraction; HFpEF indicates heart failure with preserved ejection fraction; MACE indicates Major Adverse Cardiovascular Events. AF indicates atrial fibrillation. n/N indicates number of events (n) over the total number of patients included in the analysis (N).

|  | **TTS**  **(n/N)** | **HFrEF**  **(n/N)** | **HR (95% CI)** | **TTS (n/N)** | **HFpEF**  **(n/N)** | **HR (95% CI)** |
| --- | --- | --- | --- | --- | --- | --- |
| **All-cause death** | **339/2238** | **395/2238** | **0.835 (0.722–1.005)** | **340/2224** | **391/2224** | **0.878 (0.760–1.016)** |
| **MACE** | **157/1640** | **121/1331** | **1.026 (0.810–1.301)** | **157/1627** | **142/1454** | **1.001 (0.798–1.256)** |
| **Acute Heart failure** | **78/1352** | **128/873** | **0.467 (0.409–0.533)** | **79/1340** | **120/1104** | **0.629 (0.548–0.721)** |
| **Malignant Arrhythmias** | **46/2021** | **66/2114** | **0.702 (0.482–1.023)** | **46/2012** | **59/2159** | **0.838 (0.570–1.232)** |
| **Ventricular Arrhythmias** | **47/2010** | **90/1961** | **0.490 (0.344–0.697)** | **47/1998** | **66/2068** | **0.742 (0.511–1.079)** |
| **New Onset AF** | **93/1584** | **133/1558** | **0.681 (0.523–0.888)** | **93/1571** | **129/1508** | **0.700 (0.536–0.913)** |
| **Myocardial Infarction** | **233/2238** | **342/2238** | **0.634 (0.537–0.749)** | **233/2224** | **259/2224** | **0.898 (0.753–1.072)** |
| **Stroke** | **65/1849** | **78/1863** | **0.810 (0.583–1.126)** | **65/1836** | **73/1850** | **0.891 (0.638–1.245)** |

**Supplementary Table S6.** Risks of primary and secondary outcomes in patients with TTS compared to those whit HF in clinically relevant subgroups of patients (age ≥ 65 years; < 65 years). HRs are reported with 95% confidence intervals shown in parentheses. TTS indicates Takotsubo; HF indicates Heart failure; MACE indicates Major Adverse Cardiovascular Events; AF indicates Atrial fibrillation. n/N indicates number of events (n) over the total number of patients included in the analysis (N).

|  | **TTS ≥ 65 yrs**  **(n/N)** | **HF ≥ 65 yrs**  **(n/N)** | **HR (95% CI)** | **TTS < 65 yrs**  **(n/N)** | **HF < 65 yrs**  **(n/N)** | **HR (95% CI)** |
| --- | --- | --- | --- | --- | --- | --- |
| **All-cause death** | **289/1455** | **328/1455** | **0.851 (0.727, 1.038)** | **51/785** | **87/785** | **0.589 (0.417, 1.005)** |
| **MACE** | **113/1023** | **93/860** | **0.995 (0.756, 1.309)** | **44/618** | **28/522** | **1.378 (0.858, 2.214)** |
| **Acute Heart failure** | **66/840** | **89/808** | **0.703 (0.597, 0.828)** | **13/513** | **40/463** | **0.503 (0.424, 0.620)** |
| **Malignant Arrhythmias** | **31/1357** | **36/1395** | **0.856 (0.529, 1.383)** | **15/668** | **31/735** | **0.525 (0.284, 0.973)** |
| **Ventricular Arrhythmias** | **33/1308** | **54/1336** | **0.600 (0.389, 0.925)** | **23/632** | **47/662** | **0.487 (0.296, 0.803)** |
| **New Onset AF** | **72/896** | **108/916** | **0.645 (0.478, 0.869)** | **21/691** | **30/704** | **0.717 (0.410, 1.252)** |
| **Myocardial Infarction** | **174/1455** | **194/1455** | **0.822 (0.666, 1.014)** | **59/785** | **87/785** | **0.670 (0.481, 1.063)** |
| **Stroke** | **45/1186** | **47/1164** | **0.900 (0.598, 1.355)** | **20/6564** | **19/675** | **1.075 (0.574, 2.014)** |

|  | **TTS ≥ 65 yrs (n/N)** | **HFrEF ≥ 65 yrs (n/N)** | **HR (95% CI)** | **TTS < 65 yrs**  **(n/N)** | **HFrEF < 65 yrs**  **(n/N)** | **HR (95% CI)** |
| --- | --- | --- | --- | --- | --- | --- |
| **All-cause death** | **289/1455** | **318/1455** | **0.892 (0.760, 1.046)** | **51/785** | **75/785** | **0.685 (0.480, 0.978)** |
| **MACE** | **113/1023** | **97/833** | **0.923 (0.704, 1.211)** | **44/619** | **41/517** | **0.923 (0.603, 1.412)** |
| **Acute Heart failure** | **66/840** | **77/606** | **0.577 (0.493, 0.676)** | **13/512** | **31/304** | **0.423 (0.329, 0.546)** |
| **Malignant Arrhythmias** | **31/1357** | **32/1388** | **0.968 (0.591, 1.586)** | **15/666** | **26/723** | **0.619 (0.328, 1.168)** |
| **Ventricular Arrhythmias** | **33/1308** | **60/1290** | **0.525 (0.343, 0.803)** | **14/704** | **15/690** | **0.391 (0.211, 0.727)** |
| **New Onset AF** | **72/896** | **83/885** | **0.622 (0.450, 0.861)** | **21/690** | **32/680** | **0.645 (0.372, 1.119)** |
| **Myocardial Infarction** | **174/1455** | **236/1455** | **0.691 (0.568, 0.841)** | **59/785** | **99/785** | **0.580 (0.420, 0.800)** |
| **Stroke** | **45/1186** | **56/1210** | **0.789 (0.533, 1.169)** | **20/665** | **27/669** | **0.752 (0.422, 1.340)** |

**Supplementary Table S7.** Risks of primary and secondary outcomes in patients with TTS compared to those whit HFrEF in clinically relevant subgroups of patients (age ≥ 65 years; < 65 years). HRs are reported with 95% confidence intervals shown in parentheses. TTS indicates Takotsubo; HFrEF indicates Heart failure with reduced ejection fraction; MACE indicates Major Adverse Cardiovascular Events; AF indicates Atrial fibrillation. n/N indicates number of events (n) over the total number of patients included in the analysis (N).

**Supplementary Table S8.** Risks of primary and secondary outcomes in patients with TTS compared to those whit HFpEF in clinically relevant subgroups of patients (age ≥ 65 years; < 65 years). HRs are reported with 95% confidence intervals shown in parentheses. TTS indicates Takotsubo; HFpEF indicates Heart failure with preserved ejection fraction; MACE indicates Major Adverse Cardiovascular Events; AF indicates Atrial fibrillation. n/N indicates number of events (n) over the total number of patients included in the analysis (N).

|  | **TTS ≥ 65 yrs (n/N)** | **HFpEF ≥ 65 yrs (n/N)** | **HR (95% CI)** | **TTS < 65 yrs**  **(n/N)** | **HFpEF < 65 yrs**  **(n/N)** | **HR (95% CI)** |
| --- | --- | --- | --- | --- | --- | --- |
| **All-cause death** | **288/1452** | **329/1452** | **0.890 (0.760, 1.043)** | **49/776** | **90/776** | **0.534 (0.377, 0.757)** |
| **MACE** | **112/1020** | **96/945** | **1.123 (0.855, 1.475)** | **44/608** | **46/532** | **0.854 (0.565, 1.292)** |
| **Acute Heart failure** | **66/837** | **74/664** | **0.719 (0.604, 0.835)** | **13/504** | **33/398** | **0.549 (0.421, 0.716)** |
| **Malignant Arrhythmias** | **31/1354** | **27/1417** | **1.217 (0.726, 2.038)** | **15/659** | **24/752** | **0.690 (0.362, 1.316)** |
| **Ventricular Arrhythmias** | **33/1305** | **64/1362** | **0.546 (0.359, 0.832)** | **13/697** | **25/709** | **0.513 (0.262, 1.003)** |
| **New Onset AF** | **72/894** | **95/900** | **0.774 (0.570, 1.051)** | **21/681** | **35/689** | **0.594 (0.346, 1.020)** |
| **Myocardial Infarction** | **174/1452** | **146/1452** | **1.231 (0.988, 1.534)** | **59/776** | **87/776** | **0.655 (0.470, 0.911)** |
| **Stroke** | **44/1183** | **56/1205** | **0.802 (0.541, 1.191)** | **20/654** | **22/668** | **0.922 (0.503, 1.690)** |

**Supplementary Table S9.** Risks of primary and secondary outcomes in patients with TTS compared to those whit HF in clinically relevant subgroups of patients (presence or absence of mental disorders). HRs are reported with 95% confidence intervals shown in parentheses. TTS indicates Takotsubo; HF indicates Heart failure; MACE indicates Major Adverse Cardiovascular Events; AF indicates Atrial fibrillation. n/N indicates number of events (n) over the total number of patients included in the analysis (N).

|  | **TTS mental**  **(n/N)** | **HF mental**  **(n/N)** | **HR (95% CI)** | **TTS non mental**  **(n/N)** | **HF non mental**  **(n/N)** | **HR (95% CI)** |
| --- | --- | --- | --- | --- | --- | --- |
| **All-cause death** | **96/813** | **138/813** | **0.713 (0.549, 0.925)** | **244/1424** | **255/1424** | **0.951 (0.798–1.134)** |
| **MACE** | **56/649** | **45/532** | **1.044 (0.705, 1.545)** | **101/989** | **89/858** | **0.994 (0.747, 1.321)** |
| **Acute Heart failure** | **20/498** | **29/469** | **0.617 (0.471, 0.806)** | **59/853** | **107/783** | **0.632 (0.539, 0.742)** |
| **Malignant Arrhythmias** | **11/719** | **15/771** | **0.805 (0.370, 1.753)** | **35/1302** | **38/1365** | **0.948 (0.599, 1.500)** |
| **Ventricular Arrhythmias** | **16/730** | **22/741** | **0.752 (0.395, 1.432)** | **59/831** | **93/737** | **0.680 (0.471, 0.981)** |
| **New Onset AF** | **28/594** | **41/602** | **0.705 (0.436, 1.022)** | **65/990** | **107/993** | **0.606 (0.445, 0.824)** |
| **Myocardial Infarction** | **54/813** | **81/813** | **0.653 (0.463, 0.922)** | **179/1424** | **209/1424** | **0.837 (0.686, 0.946)** |
| **Stroke** | **27/684** | **23/679** | **1.816 (1.018, 3.869)** | **38/1163** | **53/1185** | **0.716 (0.472, 1.046)** |

**Supplementary Figure S1. Cohort construction flowchart illustrating the identification of patients with Takotsubo syndrome (TTS) and heart failure (HF) within the TriNetX Research Network.**


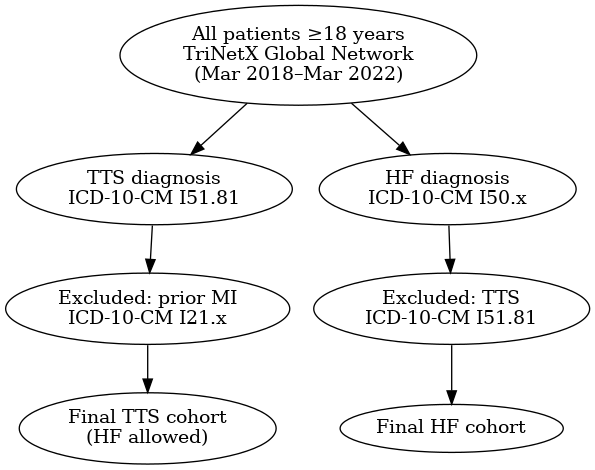

Supplement: xvag065_Supplementary_Data [file xvag065_supplementary_data.docx]
